# Untargeted metabolomics yields insight into extramammary Paget’s disease mechanisms

**DOI:** 10.3389/fonc.2024.1319819

**Published:** 2024-01-29

**Authors:** Long Jiang, Xiaoxiang Xu, Guorong Yan, Yuhao Wu, Ningyuan Xi, Yongxian Lai, Guolong Zhang, Yeqiang Liu

**Affiliations:** ^1^Department of Dermatologic Surgery, Shanghai Skin Disease Hospital, School of Medicine, Tongji University, Shanghai, China; ^2^Skin Cancer Center, Shanghai Skin Disease Hospital, School of Medicine, Tongji University, Shanghai, China; ^3^Department of Pathology, Shanghai Skin Disease Hospital, School of Medicine, Tongji University, Shanghai, China; ^4^Department of Phototherapy, Shanghai Skin Disease Hospital, School of Medicine, Tongji University, Shanghai, China; ^5^Institute of Photomedicine, School of Medicine, Tongji University, Shanghai, China

**Keywords:** extramammary Paget’s disease, untargeted metabolomics, kynurenine pathway, TDO2, IDO1

## Abstract

**Background:**

Extramammary Paget’s disease (EMPD) is a rare cutaneous malignancy, commonly affecting the external genitalia and perianal area of the elderly with unclear pathogenesis. Metabolomics provides a novel perspective for uncovering the metabolic mechanisms of a verity of cancers.

**Materials and methods:**

Here, we explored the metabolome of EMPD using an untargeted strategy. In order to further investigate the potential relationship between metabolites and gene expression, we re-analyzed the gene expression microarray data (GSE117285) using differential expression analysis and functional enrichment analyses.

**Results:**

Results showed that a total of 896 metabolites were identified and 87 metabolites including 37 upregulated and 50 downregulated significantly in EMPD were sought out. In the following feature selection analyses, four metabolites, namely, cyclopentyl fentanyl-d5, LPI 17:0, guanosine-3’,5’-cyclic monophosphate, kynurenine (KYN, high in EMPD) were identified by both random forest and support vector machine analyses. We then identified 1,079 dysfunctional genes: 646 upregulated and 433 downregulated in EMPD. Specifically, the tryptophan-degrading enzyme including indoleamine-2,3-dioxygenase-1 (*IDO1*) and tryptophan 2,3-dioxygenase (*TDO2*) were also increased. Generally, cancers exhibit a high expression of *IDO1* and *TDO2* to catabolize tryptophan, generating abundant KYN. Moreover, we also noticed the abnormal activation of sustaining proliferative signaling in EMPD.

**Conclusion:**

In conclusion, this study was the first to reveal the metabolome profile of EMPD. Our results demonstrate that *IDO1*/*TDO2*-initialized KYN metabolic pathway may play a vital role in the development and progression of EMPD, which may serve as a potential therapeutic target for treating EMPD.

## 1 Introduction

Extramammary Paget’s disease (EMPD) is a rare malignant intraepidermal adenocarcinoma that mainly affects the regions rich in apocrine glands, such as the external genitalia, perianal, inguinal, and axilla (1). Globally, the incidence of EMPD was estimated at 0.1–2.4/10^6^/year (2). Specifically, the prevalence is four times higher in the Asian population than in the worldwide (2). EMPD seems to show obvious racial differences, as it is common in menopausal women in Caucasians, while the incidence is significantly higher in males in China, with a male-to-female ratio of about 8:1 (3). In the early stage of EMPD, the skin lesions have no distinct specificity and are easily confused with non-tumor skin diseases such as eczema and jock itch, and the misdiagnosis rate is high. In 2015, a retrospective study of 246 Chinese male patients with EMPD found that almost all patients were not diagnosed in time, and the average time to diagnosis was 43.2 months after the appearance of the skin lesion (4). Early diagnosis is imperative because the diagnosis is frequently delayed and there is a high incidence of related invasive disorders.

Surgical resection is still the preferred approach for EMPD treatment. However, due to the unclear tumor boundary and affected mucosal regions (e.g., anorectal, urethra, vagina), EMPD is hard to be resected completely. Thus, most EMPD patients experience recurrence 2–3 years after surgery, and the recurrence rate is almost 60% (1). Additionally, those patients with special and huge skin lesions after surgery require skin transplantation and tissue reconstruction, often suffer postoperative appearance and structure function. Furthermore, approximately 20% of EMPD are invasive (5), the incidence of proximal lymph node and distant metastases in invasive EMPD ranges from 34%–61%, and the 5-year survival rate after distant metastases is only 7% (6, 7).

These difficulties mentioned above are caused by the unclear pathogenesis of EMPD. Therefore, uncovering the pathogenesis of EMPD will facilitate the identification of effective therapeutic interventions to repress or eliminate it in the early stage. Most studies mainly focused on evaluating the clinicopathological characteristics of EMPD by immunohistochemical (IHC) staining, and the pathogenesis has not been revealed (8, 9). With the sequencing technology development, more and more researchers employed whole-exome sequencing (WES) and gene expression array or RNA-sequencing approaches to dissect the mechanism of EMPD. Several genes, such as *KMT2C* (10) with high mutation, *FOXA1* (11), and *NEAT1* (12) were revealed as potential oncogenic genes for EMPD. However, there is little knowledge about the distortion of metabolism in EMPD. Metabolomics is a new omics after genomics, transcriptomics, and proteomics, which represents all of the low-molecular metabolites and it is an important reflection of various disorders (13). Thus, the metabolic signature of EMPD needs to be investigated.

In the present study, we aim to explore the metabolome of EMPD and identify the metabolic marker of EMPD, which will facilitate the illumination of the pathogenesis of EMPD and the diagnosis and treatment of EMPD from the perspective of metabolomics.

## 2 Materials and methods

### 2.1 Sample collection and preparation

This study was reviewed and approved by the Medical Ethics Committee of Shanghai Skin Disease Hospital, and all participants provided were consented preoperatively to enroll in this study with the understanding that it might be published. All samples were used according to the ethical standards. A total of six EMPD patients were enrolled in this study, and all diagnoses of EMPD were verified by hematoxylin and eosin (HE) staining and IHC staining before the definitive surgical procedure according to our previous staining protocol (14). All the IHC staining antibodies were purchased from Mai Xin Biotechnology Development Co., Ltd., including pan cytokeratin (CK, MAB-0050), CK7 (MAB-0828), carcinoembryonic antigen (CEA, MAB-0852), and epithelial membrane antigen (EMA, Kit-0011). Additionally, patient-matched normal adjacent skin tissue was taken from the area around the EMPD lesion, which was determined by two clinicians to be normal skin, and part of the tissue was subjected to HE staining to confirm that it was normal tissue. All these six patients were males with ages ranging from 62 to 82 (71.5 ± 7.92) years old. Then, the EMPD tumor sample and patient-matched normal adjacent skin sample were collected during surgical resection and immediately quickly frozen in liquid nitrogen and stored at −80°C refrigerator for following metabolic experiment.

Then, a total of 100 mg of each sample was grounded in liquid nitrogen and the homogenate was transferred into the EP tube with 500 μL prechilled 80% methanol to resuspend by well vortex. The samples were placed on ice for 5 min and then were centrifuged at 15,000*g* for 20 min at 4°C. Then, supernatant for each sample was diluted by the liquid chromatography-mass spectrometry (LC-MS) grade water to final concentration containing 53% methanol. Subsequently, the samples were transferred to a new tube and then were centrifuged at 15,000*g* for 20 min at 4°C. To generate the quality control (QC) sample, 20 mL of supernatant from each sample was mixed into a QC sample. Finally, the supernatant of each sample was injected into the liquid chromatography tandem-mass spectrometry (LC-MS/MS) system (15).

### 2.2 Untargeted metabolome experiment

Ultra high performance liquid chromatography tandem-mass spectrometry (UHPLC-MS/MS) analysis was performed on the Vanquish UHPLC system (Thermo Fisher Scientific, Massachusetts, USA) coupled with an Orbitrap Q Exactive™ HF mass spectrometer (Thermo Fisher Scientific, Massachusetts, USA). In detail, chromatographic separation was conducted in Vanquish UHPLC system equipped with a Hypersil GOLD columns (100 mm × 2.1 mm, 1.9 μm) using a 17-min linear gradient at a flow rate of 0.2 mL/min. The eluents for the positive polarity mode were eluent A (0.1% FA in water) and eluent B (methanol). The eluents for the negative polarity mode were eluent A (5 mM ammonium acetate, pH 9.0) and eluent B (methanol). The solvent linear gradient was set as follows: 2% B, 1.5 min; 2%−100% B, 12.0 min; 100% B, 14.0 min; 100%−2% B, 14.1 min; 2% B, 17 min. The ESI-MSn experiments were executed on a Q ExactiveTM HF mass spectrometer in positive/negative polarity mode with spray voltage of 3.2 kV. Sheath gas and auxiliary gas flow e were set at 40 and 10 arbitrary units, respectively, and the capillary temperature was 320°C. The analyzer scanned over a mass range of 100 m/z–15,000 m/z for full scan at a mass resolution of 70,000. Finally, data-dependent acquisition MS/MS experiments were performed.

### 2.3 Data preprocessing and metabolite identification

The raw data generated by UHPLC-MS/MS platform was processed using the Compound Discoverer software (v3.1, Thermo Fisher Scientific, Massachusetts, USA) to perform peak alignment, peak picking, and quantitation. The main parameters were set as follows: 0.2 min for retention time tolerance, 5 ppm for actual mass tolerance, 30% for signal intensity tolerance, 3 for signal/noise ratio, and minimum intensity. After that, peak intensities were normalized to the total spectral intensity. Then, the normalized data were used to predict the molecular formula based on additive ions, molecular ion peaks, and fragment ions, which were further matched with the mzCloud, mzVault, and MassList database to obtain the accurate qualitative and relative quantitative results of metabolites.

### 2.4 Bioinformatics analyses

All metabolites were annotated using the Kyoto Encyclopedia of Genes and Genomes (KEGG) database, HMDB database, and LIPIDMaps database. Data normalization, principal components analysis (PCA), partial least squares discriminant analysis (PLS-DA), random forest (RF) and support vector machine (SVM) were performed using *MetaboAnalystR* R-package (16). Univariate analysis (*t*-test) was used to calculate the differential metabolites. Any metabolites with the value of value importance in projection (VIP) > 1, *P* < 0.05 and | log_2_ (fold change) | > 1 were considered to be differential metabolites. To view the metabolites content, the metabolites data were normalized as z-scores and visualized by *pheatmap* R-package. The differential metabolites were used to conduct an over-representation analysis enrichment analysis, and enriched KEGG pathways with *P* < 0.05 were considered as statistically significant.

### 2.5 Gene expression microarray analysis

To link the potential relationship between metabolome and transcriptome in EMPD, we described the gene expression profile of EMPD using a public microarray dataset with the accession code of GSE117285 from the Gene Expression Omnibus database (17). Specifically, gene expressions of eight samples, including four normal skins and four EMPD patients, were obtained from Agilent-039494 Sure-Print G3 Human GE v2 8 × 60K microarray. After QC, differentially expressed genes (DEGs) identification was conducted using *limma* R-package with log_2_ transformed expression signal matrix. *P*-value was adjusted by false discovery rate. Gene with | log_2_ (fold change) | > 1 and an adjusted *P* < 0.05 was considered as a differential expression.

Then, the DEGs were used for Gene Ontology (GO) functional annotation, regarding biological process (BP), cellular component (CC), and molecular function (MF) using *clusterProfiler* R-package (18). A term with *P* < 0.05 was considered statistically significant. Gene set enrichment analysis (GSEA) was conducted based on the results of the DEG analysis using the *clusterProfiler* R-package, and C2-curated gene sets of KEGG pathway for human (v7.2) was downloaded from MSigDB (19). The gene list was ranked according to the log_2_ (fold change) and used as an input for the GSEA. *P*-value was adjusted by Benjamini and Hochberg method and adjusted *P* < 0.05 was considered as statistically significant. All the results were visualized in R software with different packages.

### 2.6 Metabolite content validation

In order to validate the metabolome result, we employed another independent HPLC-MS instrument to validate the differential metabolite in EMPD and normal skin samples, which was performed by Wuhan Servicebio Technology Co., LTD according to a standard protocol. Briefly, 100 μL of each sample was prepared, then added into 900 μL of methanol, and centrifuged at 13,000 rpm for 10 min at 4°C. The supernatant was collected for HPLC-MS analysis with using Thermo TSQ Quantum (Thermo Fisher Scientific, Germany) and Thermo UltiMate 3000 RS (Thermo Fisher Scientific, Germany). Finally, data were analyzed by Xcalibur (Thermo Fisher Scientific, Germany).

## 3 Results

### 3.1 Morphological characteristics of EMPD

**Figure 1:**
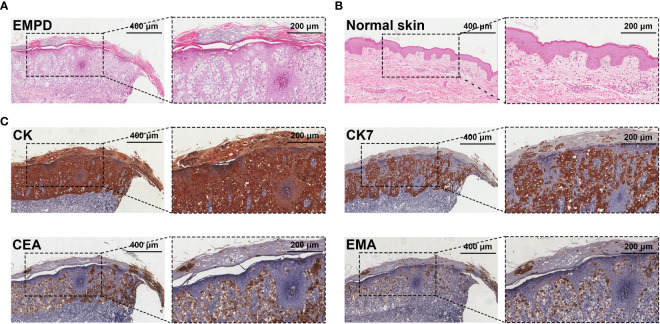
Pathological examination of EMPD. **(A)** HE staining shows Paget’s cells in the epidermis of a resected EMPD specimen. Paget’s cells are characteristic, with large nuclei and abundant clear cytoplasm on haematoxylin and eosin staining. **(B)** HE staining of normal skin. **(C)** IHC staining of CK, CK7, CEA, and EMA for the diagnoses of EMPD.

All EMPD patients were confirmed by HE and IHC before surgery. The key pathological characteristic was the presence of Paget’s cells in the epidermis (**Figure 1**). Paget’s cells exhibited a clear staining with abundant cytoplasm and large nuclei and are often associated with mitosis. IHC staining showed high expression of CEA, EMA, and CK7 in Paget’s cells. Malignant cells sometimes infiltrated the hair follicles and sweat gland ducts.

### 3.2 Metabolome of EMPD

**Figure 2:**
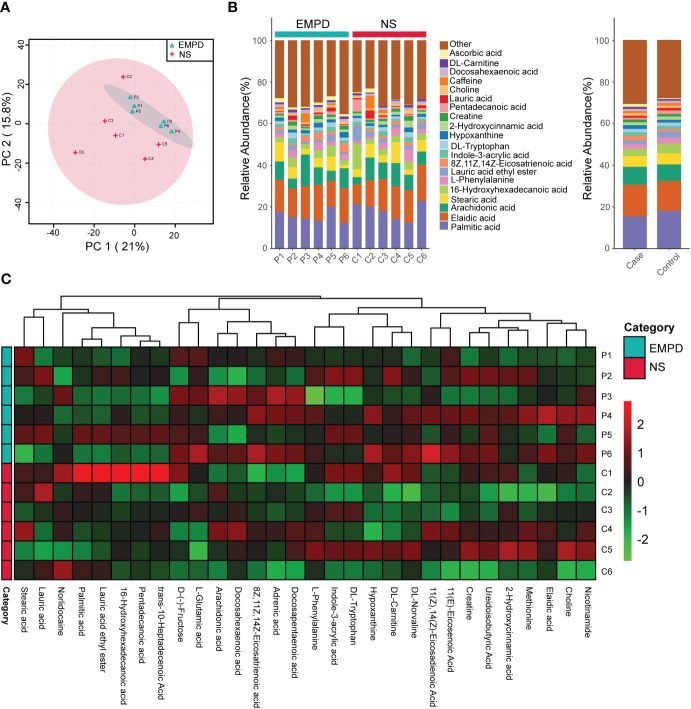
Metabolic profile EMPD. **(A)** Principal component analysis (PCA) of the metabolome of the studied samples. **(B)** Metabolites compositions. **(C)** Heatmap of the top 30 common metabolites. NS, normal skin.

To clarify the profile of local lipid metabolism in EMPDs, we used non-targeted metabolome analysis to detect metabolites. A total of 896 metabolites were identified. PCA showed that a larger heterogeneity was found in the control group than in the EMPD group, as the samples from EMPD group gathered very well (**Figure 2A**). Then, we described the metabolic profile using a stacked histogram with calculating the percentage of each metabolite in each individual. The top 20 metabolites including Palmitic acid, Elaidic acid, Arachidonic acid, Stearic acid, 16-Hydroxyhexadecanoic acid, L-Phenylallaine, Lauric acid ethyl ester, 8Z,11Z,14Z-Eicosatrienoic acid, Indole-3-acrylic acid, DL-tryptophan, and so on (**Figure 2B**). The metabolites except the top 20 were grouped into the other class. Similarly, the grouped percentage of each metabolite for these two groups was also showed (**Figure 2B**). The heatmap showed the top 30 metabolites across different samples (**Figure 2C**). Generally, there was no significant difference for the major metabolites between the control and the EMPD group.

### 3.3 Differential metabolites in EMPD by univariate analysis

**Figure 3:**
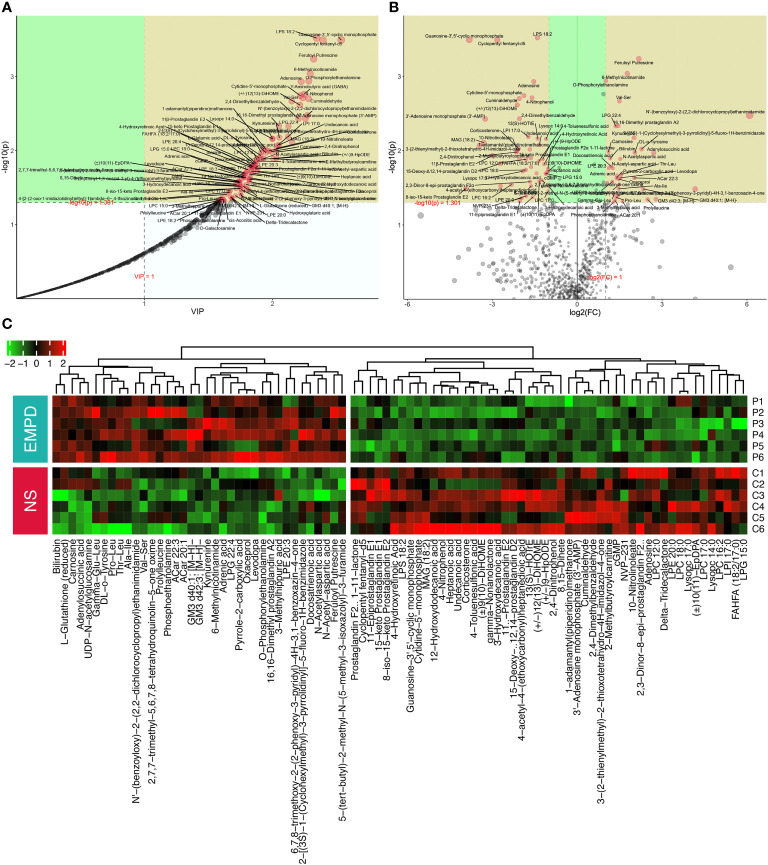
Differential metabolites identification. **(A)** PLS-DA metabolite importance map. each point represents a metabolite, the *x*-axis is the value importance in projection (VIP), and the *y*-axis is the *P*-value (calculated by *t*-test) adjusted by FDR. **(B)** Volcano plot for differential metabolites identification between normal skin and EMPD. **(C)** Heatmap of the identified differential metabolites. NS, normal skin.

The PLS-DA model was used to calculate the importance of each metabolite defined by VIP value (**Figure 3A**). In general, the VIP value of a differential metabolite should > 1. Univariate analysis was used to detect the differential metabolites between these two groups. We quantified the change of each metabolite by calculating the fold change (FC) of the normalized metabolite content and identified the significance with the *P*-value by *t*-test. A total of 87 metabolites including 37 upregulated and 50 downregulated significantly in EMPD were identified (**Figure 3B**), which should be studied in the further research. Specifically, feruloyl putrescine was the most significantly upregulated metabolite in EMPD (FC = 4.52, VIP = 2.32), followed by 6-Methylnicotinamide (FC = 3.41, VIP = 2.29), and O-Phosphorylethanolamine (FC = 2.02, VIP = 2.28). Regarding the downregulated metabolites, lipopolysaccharide (LPS) 18:2 was the most downregulated one (FC = 0.38, VIP = 2.34), followed by Guanosine-3’,5’-cyclic monophosphate (FC = 0.07, VIP = 2.54), and Cyclopentyl fentanyl-d5 (FC = 0.14, VIP = 2.39). To visually show the contents of 87 differential metabolites among these 12 samples, unsupervised cluster method and heatmap were used. Two distinct clusters in each group were spited automatically, representing downregulated and upregulated metabolites, respectively (**Figure 3C**).

### 3.4 Feature selection in EMPD by machine learning

**Figure 4:**
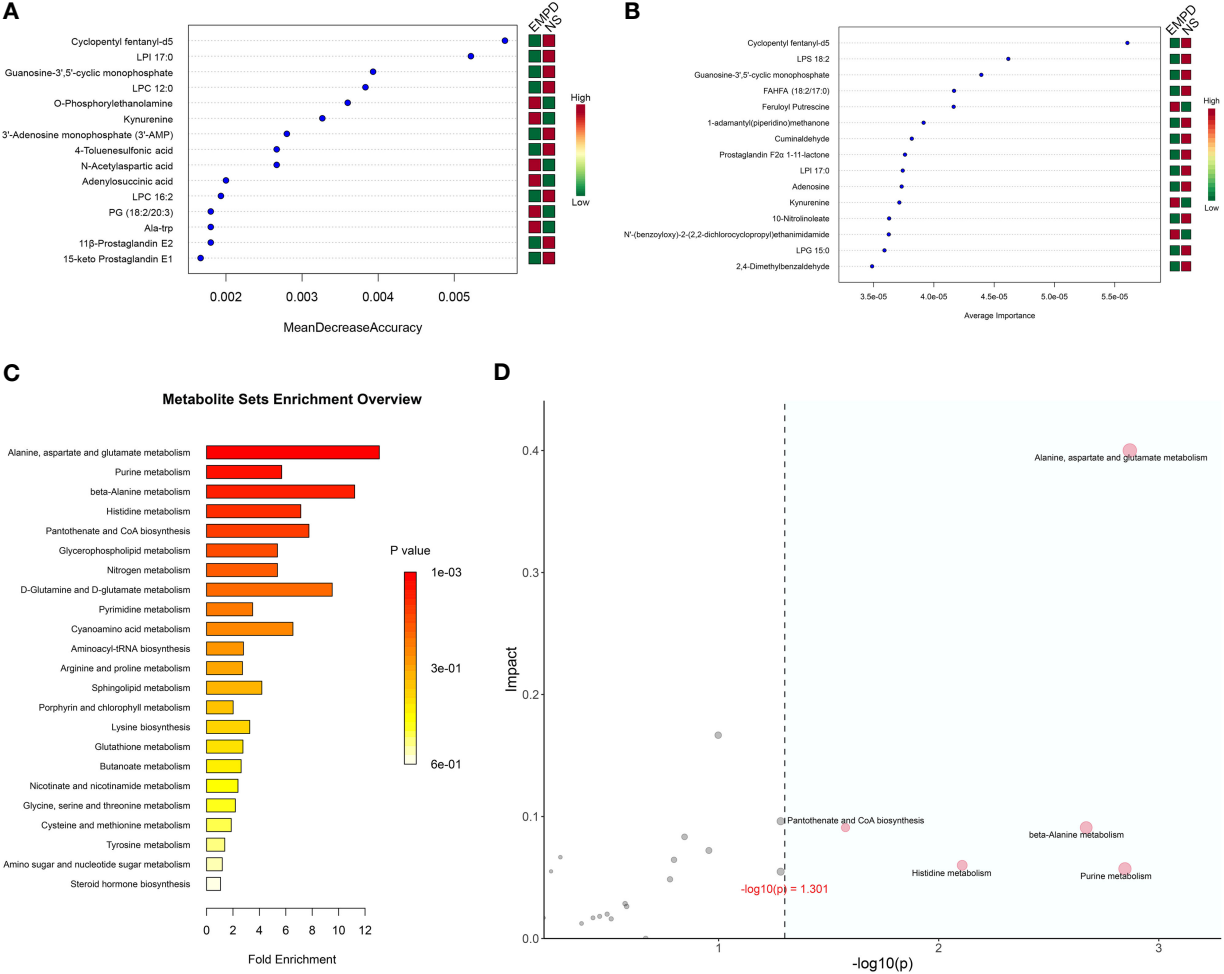
EMPD-specific metabolites selection and enrichment analysis. **(A)** The top 15 most important metabolites identified by random forests. **(B)** The top 15 most important metabolites identified by support vector machine. **(C)** Enrichment analysis. The *x*-axis is the fold enrichment factor, which is calculated as metabolite ratio/background ratio. **(D)** Topological analysis. The *x*-axis is the *P*-value, and the blue area is significant (*P* < 0.05); the *y*-axis is the topological analysis impact. NS, normal skin.

To further identified the EMPD-specific features, machine learning methods including RF and SVM were used. The most 15 important metabolites for distinguish EMPD from the control were identified by these two methods (**Figures 4A, B**). For RF analysis, the most 15 important metabolites were Cyclopentyl fentanyl-d5, LPI 17:0, Guanosine-3’,5’-cyclic monophosphate, LPC 12:0, O-Phosphorylethanolamine, Kynurenine (KYN), 3’-Adenosine monophosphate, 4-Toluenesulfonic acid, N-Acetylaspartic acid, Adenylosuccinic acid, LPC 16:2, 11β-Prostaglandin E2, Ala-trp, and PG (18:2/20:3) (**Figure 4A**). In the SVM analysis, the most 15 important metabolites were Cyclopentyl fentanyl-d5, LPS 18:2, Guanosine-3’,5’-cyclic monophosphate, FAHFA (18:2/17:0), Feruloyl Putrescine, 1-adamantyl (piperidino) methanone, Cuminaldehyde, Prostaglandin F2α 1-11-lactone, LPI 17:0, Adenosine, KYN, 10-Nitrolinoleate, N’-(benzoyloxy)-2-(2,2-dichlorocyclopropyl) ethanimidamide, LPG 15:0, and 2,4-Dimethylbenzaldehyde (**Figure 4B**). However, only four of 15 metabolites were shared by RF and SVM analyses, including Cyclopentyl fentanyl-d5 (high in control), LPI 17:0 (high in control), Guanosine-3’,5’-cyclic monophosphate (high in control), KYN (high in EMPD). All of these four metabolites were differential between EMPD and the control group.

### 3.5 Metabolic pathway enrichment analysis

As lots of differential metabolites were identified between EMPD and the control group, KEGG enrichment analysis was performed to explore the potential biological pathways that may play a crucial role in the pathogenesis of EMPD. A total of five significant pathways were enriched including alanine, aspartate and glutamate metabolism, purine metabolism, beta-alanine metabolism, histidine metabolism, and pantothenate and CoA biosynthesis (**Figure 4C**). Topological analysis can assess the impact of the metabolite in the significantly enriched metabolic pathway. Therefore, we combined this analysis to determine whether a metabolic pathway plays a key role in the progression of EMPD. Similarly, alanine, aspartate, and glutamate metabolism were determined as the most impact pathway in the current study (**Figure 4D**).

**Figure 5:**
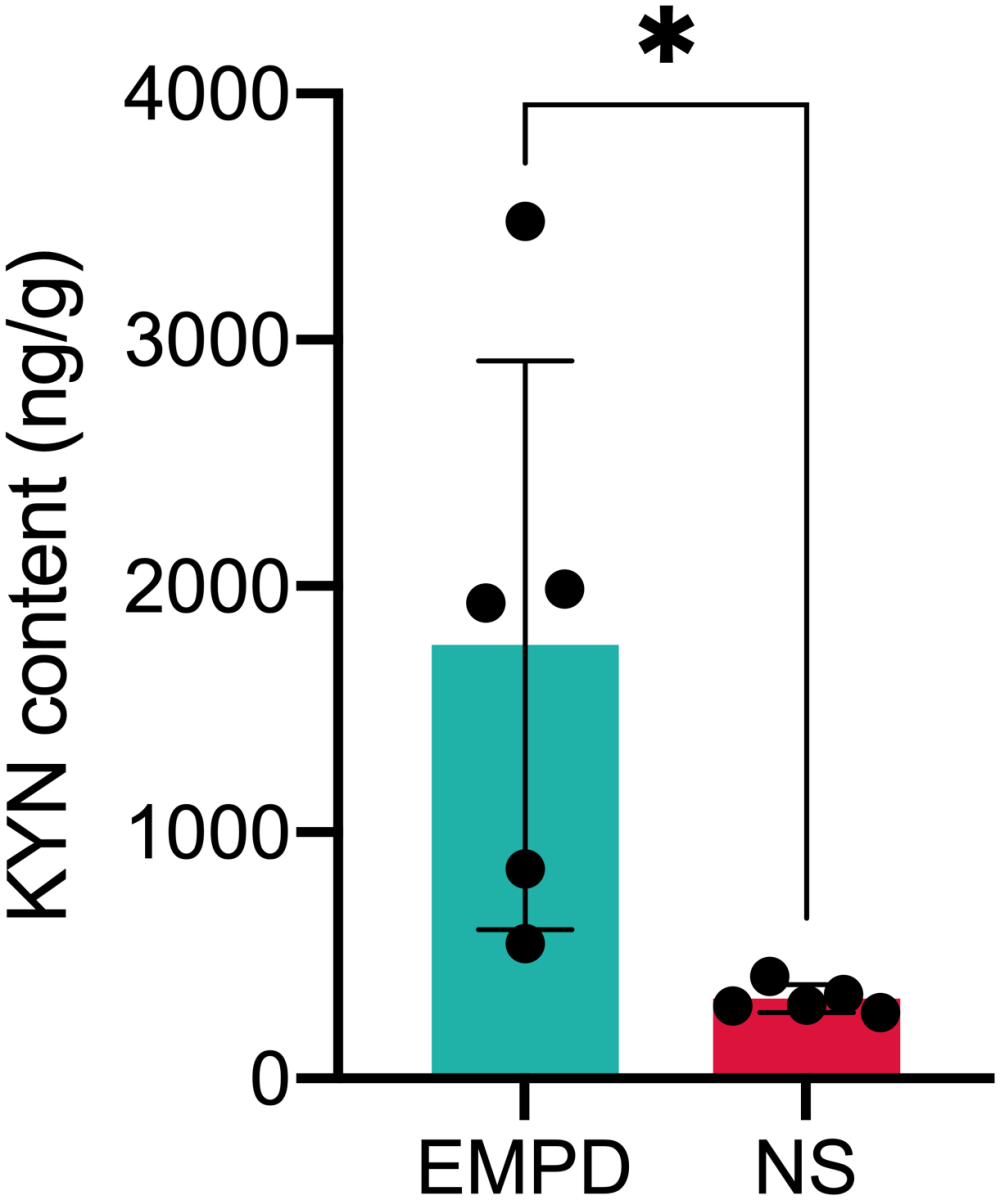
Validation of KYN content by HPLC-MS instrument. *P < 0.05 indicates the significant difference; NS, normal skin.

To further validate the differential metabolite, we used another independent HPLC-MS instrument to validate the differential metabolite in EMPD and normal skin samples. As shown in **Figure 5**, the KYN content was significantly enriched in EMPD (*P* < 0.05).

### 3.6 Changed gene expression profile of EMPD

**Figure 6:**
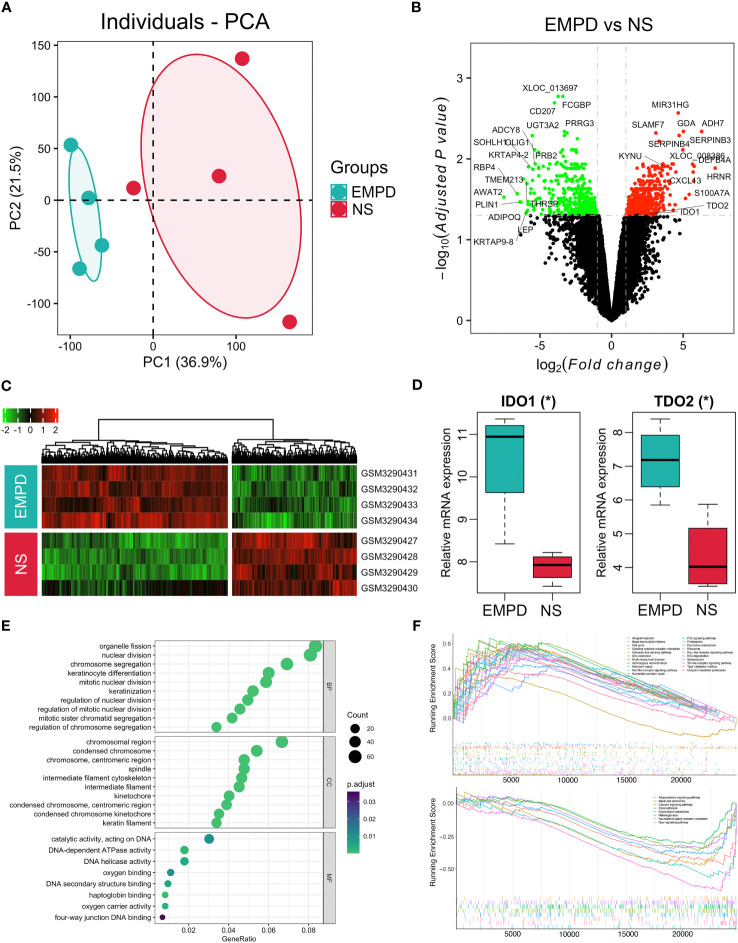
Dysfunctional gene expressions in EMPD from GSE117285. **(A)** Principal component analysis (PCA) of the gene expression matrix. **(B)** Volcano plot. **(C)** Heatmap for DEGs in all samples. **(D)** Boxplot for *IDO1* and *TDO2* expression between EMPD and the control group. **(E)** Gene Ontology analysis. BP, CC, and MF are biological process, cellular component, and molecular function, respectively. **(F)** Gene set enrichment analysis. The fold change of each gene was calculated from EMPD versus control and ordered for GSEA analysis. *P*-value was adjusted by Benjamini–Hochberg method. *P < 0.05 indicates the significant difference; NS, normal skin.

In order to further investigate the potential relationship between metabolites and gene expression, we re-analyzed the gene expression microarray data (GSE117285), which containing four EMPD patients and four normal skins. The PCA result showed that these two groups were clustered very well, indicating a distinct difference between EMPD and normal skin at the transcriptomic level (**Figure 6A**). A total of 1,079 DEGs including 646 upregulated and 433 downregulated in the EMPD patients (**Figure 6B**). All the 1,079 DEGs were presented by gene expression heatmap (**Figure 6C**). Specifically, five chemokines or their receptors including *CXCL1*, *CXCL2*, *CXCL9*, *CXCL13*, and *CXCR6* were significantly upregulated in EMPD. In addition, the tryptophan-degrading enzyme including indoleamine-2,3-dioxygenase-1 (*IDO1*) and tryptophan 2,3-dioxygenase (*TDO2*) were also increased (**Figure 6D**). Interestingly, we also observed an increase of KYN, generated by *IDO1* or *TDO2* in EMPD.

We further employed gene functional annotation to investigate the dysfunctional pathway in EMPD. The top 10 significantly enriched terms of GO enrichment analysis showed that in the BP level, the DEGs involved in organelle fission, nuclear division, keratinocyte differentiation, mitotic nuclear division, and so on (**Figure 6E**). Regarding the CC level, the DEGs were enriched in chromosome region (**Figure 6E**). In terms of the MF level, the DEGs were mainly concentrated in the catalytic activity, DNA-dependent ATPase activity, and DNA helicase activity (**Figure 6E**). Furthermore, we identified a series of KEGG pathway that activated or inhibited in EMPD by the GSEA. Specifically, a total of 21 pathways were activated in EMPD, including cell cycle, spliceosome, TLR signaling pathway, P53 signaling pathway, RNA degradation, basal transcription factors, DNA replication, and mismatch repair (**Figure 6F**). Meanwhile, eight pathways including glycerolipid metabolism, PPAR signaling pathway, adipocytokine signaling pathway, melanogenesis, calcium signaling pathway, focal adhesion, neuroactive ligand receptor interaction, and basal cell carcinoma were inhibited in EMPD (**Figure 6F**). However, although the tryptophan metabolic pathway was not significantly identified by GSEA, this pathway showed an activated trend with a positive enrichment score (0.38, adjusted *P* = 0.44). Collectively, both metabolomic and transcriptomic results indicate that tryptophan metabolic pathway may involve in the development of EMPD.

## 4 Discussion

EMPD is considered a rare malignant cutaneous carcinoma with poorly outcomes characterized with high recurrence rate. Clinically, pruritic erythematous plaque on local skin is the more frequent initial clinical presentation of EMPD (20), which needs to be differentiated from other chronic dermatitis. The diagnosis of EMPD is frequently delayed, causing older age of patients at diagnosis, which results in decreased survival (5). Thus, a series of studies focused on the dissection of EMPD pathogenesis of EMPD and biomarkers identification such as neutrophil-to-lymphocyte ratio (21). Metabolomics is becoming a powerful omics tool, which has been successfully applied to various cutaneous carcinoma including melanoma (22), cutaneous squamous cell carcinoma (23), and basal cell carcinoma (24). In the present study, we investigated the metabolic signature in EMPD by untargeted metabolomics. Although the major metabolites were not significant difference between EMPD and the paired normal skin, a total of 87 differential metabolites were still identified, which may contribute to the progression of EMPD.

In the present study, we found KYN was significantly elevated in the EMPD, and its importance was confirmed by RF and SM analyses, indicating that KYN may exert crucial role in the EMPD progression. KYN is the carboxylated product derived from the oxidative cleavage of the essential amino acid tryptophan (TRY) (25). Furthermore, The KYN pathway, initiated by indole-amine-2,3-dioxygenase 1/2 (*IDO1*/*2*) or tryptophan-2,3-dioxgenase (*TDO2*), has been considered as the leading TRY catabolic route in humans (26). Interestingly, we also observed the elevated expression of *IDO1* and *TDO2* by re-analyzed the public gene expression data (GSE117285). Accumulated evidences have reported that the activated KYN pathway has been positively correlated with the bad prognosis of different kinds of tumors including colorectal cancer (27), breast cancer (28), and melanoma (29).

KYN is the ligand of aryl hydrocarbon receptor (AHR), which is a transcription factor whose targets regulate various BPs, including angiogenesis, lipid metabolism, cell motility, and immune modulation (30–32). Therefore, cancer trends showed a high expression of *IDO1* and/or *TDO2* to catabolize TRY, generating abundant KYN that further activates AHR and its targets for the sake of tumor progression (33, 34). The KYN-AHR axis promotes the malignant phenotype of cancer cells, in particular, cancer cell motility (35). Furthermore, KYN-AHR signaling builds an immunosuppressive microenvironment through inducing regulatory T cells differentiation and recruiting immunosuppressive tumor-associated macrophages (36, 37), together with upregulating programmed cell death protein 1 expression in CD8^+^ T cells and enticing its death (28, 38). Interestingly, there is a study that has reported the action of AHR pathway in EMPD. Sato et al. has proved that the metabolites produced by *Malassezia* serve as AHR ligands to activate the AHR pathway and induce the Th17 immune response (39).

Except for the increased expression of *IDO1* and *TDO2* in EMPD, we also noticed various dysfunctional pathways. More than 20 pathways were activated in EMPD, including cell cycle, basal transcription factors, and DNA replication, indicating that a sustaining proliferative signaling was activated. Furthermore, P53 signaling pathway and mismatch repair pathway were also activated. One WES study showed that TP53 is highly mutated in EMPD, indicating that its tumor suppressor function may be compromised (40), causing tumor cells to escape from apoptosis. Taken together, the increased KYN and *IDO1*/*TDO2* indicate that TRY metabolic pathway initialized by *IDO1*/*TDO2* may exert import role in the progression of EMPD and paired metabolomic and transcriptomic analyses should be performed to confirm our findings in a large EMPD samples.

## 5 Conclusions

In summary, we explore the metabolome and transcriptome of EMPD in the present study. Both KYN and *IDO1*/*TDO2* were elevated in EMPD, demonstrating that KYN metabolic pathway may play a vital role in the development and progression of EMPD. Our findings provide basic evidence that *IDO1*/*TDO2* may be an important molecule participating in the aberrant activation of TRY metabolic pathway and thus may serve as a potential therapeutic target for treating EMPD.

## Data availability statement

The data presented in the study are deposited in the Metabolites repository, accession number MTBLS8797, and the gene expression microarray dataset was downloaded from the Gene Expression Omnibus (GEO) repository, accession number GSE117285.

## Ethics statement

The studies involving humans were approved by Medical Ethics Committee of Shanghai Skin Disease Hospital. The studies were conducted in accordance with the local legislation and institutional requirements. The participants provided their written informed consent to participate in this study.

## Author contributions

LJ: Data curation, Formal analysis, Methodology, Validation, Writing – original draft. XX: Funding acquisition, Writing – original draft. GY: Formal analysis, Validation, Visualization, Writing – review & editing. YW: Investigation, Writing – review & editing. NX: Investigation, Methodology, Writing – review & editing. YXL: Conceptualization, Project administration, Resources, Supervision, Writing – review & editing. GZ: Conceptualization, Funding acquisition, Project administration, Resources, Supervision, Writing – review & editing. YQL: Conceptualization, Funding acquisition, Project administration, Resources, Supervision, Writing – review & editing.
